# Methicillin-sensitive *Staph. aureus* (MSSA) and methicillin-resistant *Staph. aureus* (MRSA) bacteraemia decline simulaneously

**DOI:** 10.1186/1753-6561-5-S6-P232

**Published:** 2011-06-29

**Authors:** R Coello, ET Brannigan, J Wilson, M Richards, S Hassall, A Jepson, A Holmes

**Affiliations:** 1 Infection Prevention and Control, Imperial College Healthcare NHS Trust, London, UK; 2 Infectious Diseases; Infection Prevention Control, Imperial College Healthcare NHS Trust, London, UK; 3 Microbiology, Imperial College Healthcare NHS Trust, London, UK

## Introduction / objectives

The recent decline in MRSA infection is well recognised but the contribution of hospital-acquired (HA) versus community-acquired (CA) is not always clear, nor if MRSA has substituted for MSSA or is an additional burden. We examined our experience with *S.aureus* bacteraemia (SAB).

## Methods

From April 2008 to March 2010, at Imperial College Healthcare NHS Trust (ICHNT), electronic microbiological records of patients with SAB were linked with patient administration data to determine if the *S. aureus* was meticillin-sensitive or resistant, and if infection was CA or HA. SAB within 2 days of admission was defined as CA, 2 days or more after admission as HA and a new episode if more than 2 weeks between 2 positive SAB.

## Results

Of 345 SAB detected, 60% were CA and 40% HA; 250 (72%) were MSSA and 95 (28%) MRSA. Of MRSA bacteraemias, 49% were HA, but 37% of MSSA bacteraemias were HA. The rate of SAB per 100,000 bed-days had a downward trend due a decrease in both MRSA and MSSA bacteraemia. Since April 2009, the number of HA MRSA and MSSA decreased simultaneously. During the period April 2008 to March 2009, the rate of HA SAB was 19.6. In the period April 2009 to March 2010 this declined by to 11.7. Between these two periods the rates of MRSA and MSSA bacteraemia rates decreased by 1.7 (from 19.6 to 11.7), 2.1 (from 7.2 to 3.4) and 1.5 times (from 12.4-8.3).

## Conclusion

*S. aureus* as a cause of bacteraemia at ICHNT is decreasing, particularly HA cases. MRSA and MSSA bacteraemia are decreasing simultaneously, suggesting either that MRSA substituted MSSA rather than reflecting additional cases or that MRSA control measures may also have had an effect on MSSA.

## Disclosure of interest

None declared.

